# 2-Bromo-*N*-(2-hy­droxy-5-methyl­phen­yl)-2-methyl­propanamide

**DOI:** 10.1107/S1600536811033150

**Published:** 2011-08-27

**Authors:** Rodolfo Moreno-Fuquen, David E. Quintero, Fabio Zuluaga, Carlos Grande, Alan R. Kennedy

**Affiliations:** a Departamento de Química – Facultad de Ciencias, Universidad del Valle, Apartado 25360, Santiago de Cali, Colombia; b Departamento de Química – Facultad de Ciencias, Universidad ICESI, Santiago de Cali, Colombia; c WestCHEM, Department of Pure and Applied Chemistry, University of Strathclyde, 295 Cathedral Street, Glasgow G1 1XL, Scotland

## Abstract

In the title mol­ecule, C_11_H_14_BrNO_2_, there is twist between the mean plane of the amide group and the benzene ring [the C—N—C—C torsion angle is −172.1 (2)°]. The amide H atom forms an intra­molecular hydrogen bond with the Br atom. In the crystal, inter­molecular O—H⋯O and weak C—H⋯O hydrogen bonds link mol­ecules into a chain along [100].

## Related literature

For functional initiators in polymerization processes, see: Matyjaszewski & Xia (2001 ▶); Kato *et al.* (1995 ▶). For related structures, see: Moreno-Fuquen *et al.* (2011*a* ▶,*b* ▶). For hydrogen-bond graph-set motifs, see: Etter (1990 ▶).
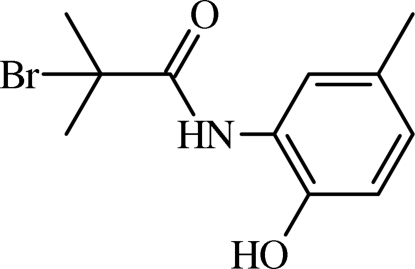


## Experimental

### 

#### Crystal data

C_11_H_14_BrNO_2_
*M* *_r_* = 272.14
Monoclinic, 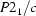

*a* = 7.4510 (2) Å
*b* = 13.8498 (4) Å
*c* = 12.8646 (4) Å
β = 116.324 (2)°
*V* = 1189.89 (6) Å^3^
*Z* = 4
Mo *K*α radiation
μ = 3.44 mm^−1^
*T* = 123 K
0.30 × 0.10 × 0.08 mm

#### Data collection

Oxford Diffraction Xcalibur E diffractometer
Absorption correction: multi-scan (*CrysAlis PRO*; Oxford Diffraction, 2009 ▶) *T*_min_ = 0.650, *T*_max_ = 1.000
5803 measured reflections
2886 independent reflections
2437 reflections with *I* > 2σ(*I*)
*R*_int_ = 0.023

#### Refinement

*R*[*F* ^2^ > 2σ(*F* ^2^)] = 0.035
*wR*(*F* ^2^) = 0.085
*S* = 1.05
2886 reflections
147 parameters
1 restraint
H atoms treated by a mixture of independent and constrained refinement
Δρ_max_ = 0.73 e Å^−3^
Δρ_min_ = −0.46 e Å^−3^

### 

Data collection: *CrysAlis CCD* (Oxford Diffraction, 2009 ▶); cell refinement: *CrysAlis CCD*; data reduction: *CrysAlis CCD*; program(s) used to solve structure: *SHELXS97* (Sheldrick, 2008 ▶); program(s) used to refine structure: *SHELXL97* (Sheldrick, 2008 ▶); molecular graphics: *ORTEP-3* (Farrugia, 1997 ▶) and *Mercury* (Macrae *et al.*, 2006 ▶); software used to prepare material for publication: *WinGX* (Farrugia, 1999 ▶) and *PARST* (Nardelli, 1995 ▶).

## Supplementary Material

Crystal structure: contains datablock(s) I, global. DOI: 10.1107/S1600536811033150/tk2780sup1.cif

Structure factors: contains datablock(s) I. DOI: 10.1107/S1600536811033150/tk2780Isup2.hkl

Supplementary material file. DOI: 10.1107/S1600536811033150/tk2780Isup3.cml

Additional supplementary materials:  crystallographic information; 3D view; checkCIF report

## Figures and Tables

**Table 1:** Hydrogen-bond geometry (Å, °)

*D*—H⋯*A*	*D*—H	H⋯*A*	*D*⋯*A*	*D*—H⋯*A*
O2—H1*H*⋯O1^i^	0.85 (1)	1.81 (1)	2.659 (2)	179 (3)
C1—H1*C*⋯O2^ii^	0.98	2.53	3.445 (3)	156
N1—H1*N*⋯Br1	0.87 (3)	2.47 (3)	3.031 (2)	123 (2)

Symmetry codes: (i) 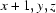; (ii) 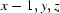.

**Fig. 1.**
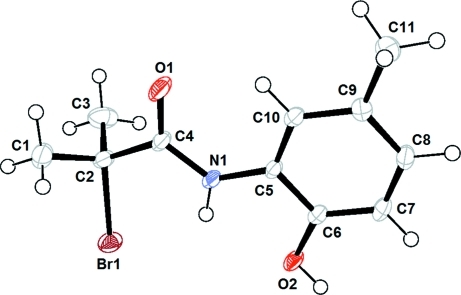
Molecular structure of (I) showing atom numbering scheme and with displacement ellipsoids drawn at the 50% probability level. H atoms are shown as spheres of arbitrary radius.

**Fig. 2.**
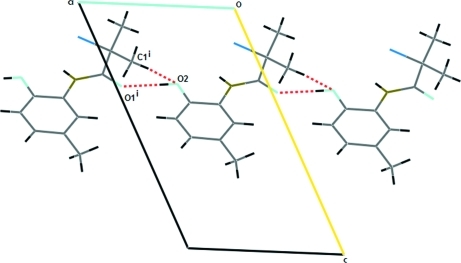
Part of the crystal structure of (I), showing the formation of a one dimensional chain along [100]. Dashed lines indicate hydrogen bonds. Symmetry code: (i) 1 + x,+y, z.

## supplementary crystallographic information

### Comment

The title compound, (I), is part of a search for new functional initiators in polymerization processes (Matyjaszewski & Xia, 2001; Kato *et al.*, 1995), which is carried out by the Polymer Group of Universidad del Valle (Moreno-Fuquen *et al.*, 2011*a*; Moreno-Fuquen *et al.*, 2011*b*). The molecular structure of (I) is shown in Fig. 1. There is a twist between the mean plane of the amide group and the benzene ring giving a C4—N1—C5—C6 torsion angle of -172.1 (2) °. This value is very different to that presented in other related systems: [12.7 (4) and -31.2 (5) °, respectively (Moreno-Fuquen *et al.*, 2011*a*; Moreno-Fuquen *et al.*, 2011*b*)]. The difference in the value of torsion angle with respect to other related systems, is probably due to the presence of the hydroxyl group in the benzene ring in (I). The crystal packing is stabilized by O—H···O and weak C—H···O intermolecular hydrogen bonds which link the molecules into one dimensional chains along [100] incorporating C(7) graphs motifs (Etter, 1990); see Table 1 and Fig. 2. Additionally, an intramolecular N—H···Br hydrogen bond is observed (Table 1).

### Experimental

2-Hydroxy-5-methyl aniline (3.512 mmol, 0.432 g), triethylamine (0.635 mmol, 0.064 g) were mixed in a 100 mL round bottom flask. Then, a solution of 2-bromo isobutryl bromide (0.807 g) in anhydrous THF (5 ml) was added drop wise, under an argon stream. The reaction was carried out in a dry bag overnight under magnetic stirring. The solid was filtered off and dichloromethane (20 ml) added to the organic phase which was washed with brine (50 ml) followed by water (10 ml). The solution was concentrated at low pressure affording colourless crystals and recrystallized from a solution of hexane and ethyl acetate  (*v*/*v* 80:20). M.pt. 385 (1) K.

### Refinement

The H-atoms were positioned geometrically [C—H = 0.95 Å for aromatic-H and C—H = 0.98 Å for methyl-H, and with *U*_iso_(H) = (1.2 and 1.5 times *U*_eq_ of the parent atom, respectively]. The hydroxyl-H1H and amide-H1N atoms were located in a difference Fourier map and were refined freely.

### Crystal data

**Table:**

C_11_H_14_BrNO_2_	*F*(000) = 552
*M**_r_* = 272.14	*D*_x_ = 1.519 Mg m^−^^3^
Monoclinic, *P*2_1_/*c*	Melting point: 385(1) K
Hall symbol: -P 2ybc	Mo *K*α radiation, λ = 0.71073 Å
*a* = 7.4510 (2) Å	Cell parameters from 3082 reflections
*b* = 13.8498 (4) Å	θ = 3.1–29.4°
*c* = 12.8646 (4) Å	µ = 3.44 mm^−^^1^
β = 116.324 (2)°	*T* = 123 K
*V* = 1189.89 (6) Å^3^	Tablet, colourless
*Z* = 4	0.30 × 0.10 × 0.08 mm

### Data collection

**Table:**

Oxford Diffraction Xcalibur E diffractometer	2886 independent reflections
Radiation source: fine-focus sealed tube	2437 reflections with *I* > 2σ(*I*)
graphite	*R*_int_ = 0.023
ω scans	θ_max_ = 29.4°, θ_min_ = 3.1°
Absorption correction: multi-scan (*CrysAlis PRO*; Oxford Diffraction, 2009)	*h* = −8→10
*T*_min_ = 0.650, *T*_max_ = 1.000	*k* = −16→19
5803 measured reflections	*l* = −16→17

### Refinement

**Table:**

Refinement on *F*^2^	Primary atom site location: structure-invariant direct methods
Least-squares matrix: full	Secondary atom site location: difference Fourier map
*R*[*F*^2^ > 2σ(*F*^2^)] = 0.035	Hydrogen site location: inferred from neighbouring sites
*wR*(*F*^2^) = 0.085	H atoms treated by a mixture of independent and constrained refinement
*S* = 1.05	*w* = 1/[σ^2^(*F*_o_^2^) + (0.0441*P*)^2^ + 0.2346*P*] where *P* = (*F*_o_^2^ + 2*F*_c_^2^)/3
2886 reflections	(Δ/σ)_max_ < 0.001
147 parameters	Δρ_max_ = 0.73 e Å^−^^3^
1 restraint	Δρ_min_ = −0.46 e Å^−^^3^

### Special details

**Table:**

Geometry. All esds (except the esd in the dihedral angle between two l.s. planes) are estimated using the full covariance matrix. The cell esds are taken into account individually in the estimation of esds in distances, angles and torsion angles; correlations between esds in cell parameters are only used when they are defined by crystal symmetry. An approximate (isotropic) treatment of cell esds is used for estimating esds involving l.s. planes.
Refinement. Refinement of F^2^ against ALL reflections. The weighted R-factor wR and goodness of fit S are based on F^2^, conventional R-factors R are based on F, with F set to zero for negative F^2^. The threshold expression of F^2^ > 2sigma(F^2^) is used only for calculating R-factors(gt) etc. and is not relevant to the choice of reflections for refinement. R-factors based on F^2^ are statistically about twice as large as those based on F, and R- factors based on ALL data will be even larger.

### Fractional atomic coordinates and isotropic or equivalent isotropic displacement parameters (Å^2^)

**Table:**

	*x*	*y*	*z*	*U*_iso_*/*U*_eq_	
Br1	0.10479 (3)	0.358681 (19)	0.14350 (2)	0.02782 (10)	
O1	−0.0362 (3)	0.54556 (15)	0.34215 (18)	0.0387 (5)	
O2	0.5930 (2)	0.53566 (12)	0.32150 (14)	0.0219 (4)	
N1	0.2461 (3)	0.51979 (14)	0.32142 (17)	0.0190 (4)	
C1	−0.1738 (4)	0.35819 (19)	0.2380 (3)	0.0317 (6)	
H1A	−0.0742	0.3209	0.3025	0.048*	
H1B	−0.2525	0.3146	0.1739	0.048*	
H1C	−0.2629	0.3916	0.2638	0.048*	
C2	−0.0677 (3)	0.43204 (18)	0.1973 (2)	0.0227 (5)	
C3	−0.2146 (4)	0.4905 (2)	0.0933 (2)	0.0375 (7)	
H3A	−0.2935	0.4466	0.0296	0.056*	
H3B	−0.1398	0.5352	0.0680	0.056*	
H3C	−0.3043	0.5272	0.1158	0.056*	
C4	0.0531 (3)	0.50331 (18)	0.2951 (2)	0.0215 (5)	
C5	0.3773 (3)	0.58845 (16)	0.39924 (19)	0.0162 (4)	
C6	0.5634 (3)	0.59619 (17)	0.39643 (19)	0.0177 (4)	
C7	0.7014 (3)	0.66329 (17)	0.4661 (2)	0.0220 (5)	
H7	0.8263	0.6696	0.4635	0.026*	
C8	0.6582 (3)	0.72175 (17)	0.5400 (2)	0.0220 (5)	
H8	0.7538	0.7681	0.5870	0.026*	
C9	0.4764 (3)	0.71337 (16)	0.54611 (19)	0.0193 (5)	
C10	0.3369 (3)	0.64603 (15)	0.4745 (2)	0.0182 (5)	
H10	0.2123	0.6396	0.4774	0.022*	
C11	0.4344 (4)	0.77409 (18)	0.6301 (2)	0.0261 (5)	
H11A	0.4697	0.7376	0.7019	0.039*	
H11B	0.2918	0.7907	0.5955	0.039*	
H11C	0.5144	0.8334	0.6476	0.039*	
H1H	0.711 (2)	0.540 (2)	0.328 (3)	0.038 (8)*	
H1N	0.297 (4)	0.4836 (19)	0.286 (2)	0.027 (7)*	

### Atomic displacement parameters (Å^2^)

**Table:**

	*U*^11^	*U*^22^	*U*^33^	*U*^12^	*U*^13^	*U*^23^
Br1	0.02120 (15)	0.03380 (17)	0.02828 (15)	0.00141 (11)	0.01082 (11)	−0.01028 (10)
O1	0.0186 (9)	0.0505 (13)	0.0535 (12)	−0.0127 (9)	0.0219 (9)	−0.0306 (10)
O2	0.0130 (8)	0.0300 (9)	0.0261 (9)	−0.0022 (7)	0.0118 (7)	−0.0037 (7)
N1	0.0128 (9)	0.0224 (10)	0.0239 (10)	−0.0013 (8)	0.0100 (8)	−0.0044 (8)
C1	0.0248 (13)	0.0322 (15)	0.0411 (16)	−0.0082 (11)	0.0174 (12)	−0.0120 (12)
C2	0.0149 (11)	0.0267 (13)	0.0256 (12)	0.0011 (10)	0.0082 (9)	−0.0070 (10)
C3	0.0273 (14)	0.0417 (17)	0.0313 (15)	0.0126 (13)	0.0021 (11)	−0.0042 (13)
C4	0.0133 (11)	0.0265 (12)	0.0261 (12)	−0.0020 (10)	0.0101 (9)	−0.0042 (10)
C5	0.0106 (10)	0.0176 (11)	0.0183 (10)	−0.0001 (9)	0.0044 (8)	0.0030 (8)
C6	0.0138 (11)	0.0207 (12)	0.0183 (11)	0.0002 (9)	0.0069 (9)	0.0034 (9)
C7	0.0143 (11)	0.0264 (13)	0.0250 (12)	−0.0049 (10)	0.0084 (9)	0.0024 (10)
C8	0.0174 (11)	0.0216 (12)	0.0230 (12)	−0.0054 (10)	0.0053 (9)	−0.0013 (9)
C9	0.0202 (11)	0.0177 (11)	0.0169 (11)	−0.0003 (10)	0.0056 (9)	0.0025 (9)
C10	0.0139 (11)	0.0206 (12)	0.0207 (11)	0.0004 (9)	0.0081 (9)	0.0024 (9)
C11	0.0277 (13)	0.0257 (13)	0.0241 (12)	−0.0011 (11)	0.0110 (10)	−0.0044 (10)

### Geometric parameters (Å, °)

**Table:**

Br1—C2	1.988 (2)	C3—H3C	0.9800
O1—C4	1.229 (3)	C5—C10	1.387 (3)
O2—C6	1.367 (3)	C5—C6	1.407 (3)
O2—H1H	0.847 (10)	C6—C7	1.381 (3)
N1—C4	1.343 (3)	C7—C8	1.391 (3)
N1—C5	1.412 (3)	C7—H7	0.9500
N1—H1N	0.87 (3)	C8—C9	1.396 (3)
C1—C2	1.520 (3)	C8—H8	0.9500
C1—H1A	0.9800	C9—C10	1.396 (3)
C1—H1B	0.9800	C9—C11	1.508 (3)
C1—H1C	0.9800	C10—H10	0.9500
C2—C3	1.531 (4)	C11—H11A	0.9800
C2—C4	1.537 (3)	C11—H11B	0.9800
C3—H3A	0.9800	C11—H11C	0.9800
C3—H3B	0.9800		
			
C6—O2—H1H	112 (2)	C10—C5—C6	119.7 (2)
C4—N1—C5	128.39 (19)	C10—C5—N1	125.84 (19)
C4—N1—H1N	115.9 (18)	C6—C5—N1	114.43 (19)
C5—N1—H1N	115.7 (18)	O2—C6—C7	124.29 (19)
C2—C1—H1A	109.5	O2—C6—C5	116.13 (19)
C2—C1—H1B	109.5	C7—C6—C5	119.6 (2)
H1A—C1—H1B	109.5	C6—C7—C8	120.2 (2)
C2—C1—H1C	109.5	C6—C7—H7	119.9
H1A—C1—H1C	109.5	C8—C7—H7	119.9
H1B—C1—H1C	109.5	C7—C8—C9	121.0 (2)
C1—C2—C3	112.2 (2)	C7—C8—H8	119.5
C1—C2—C4	110.8 (2)	C9—C8—H8	119.5
C3—C2—C4	107.9 (2)	C8—C9—C10	118.4 (2)
C1—C2—Br1	106.92 (16)	C8—C9—C11	120.6 (2)
C3—C2—Br1	106.75 (16)	C10—C9—C11	121.0 (2)
C4—C2—Br1	112.25 (14)	C5—C10—C9	121.1 (2)
C2—C3—H3A	109.5	C5—C10—H10	119.5
C2—C3—H3B	109.5	C9—C10—H10	119.5
H3A—C3—H3B	109.5	C9—C11—H11A	109.5
C2—C3—H3C	109.5	C9—C11—H11B	109.5
H3A—C3—H3C	109.5	H11A—C11—H11B	109.5
H3B—C3—H3C	109.5	C9—C11—H11C	109.5
O1—C4—N1	123.2 (2)	H11A—C11—H11C	109.5
O1—C4—C2	117.44 (19)	H11B—C11—H11C	109.5
N1—C4—C2	119.26 (19)		
			
C5—N1—C4—O1	−3.6 (4)	C10—C5—C6—C7	−2.1 (3)
C5—N1—C4—C2	173.2 (2)	N1—C5—C6—C7	177.9 (2)
C1—C2—C4—O1	−53.7 (3)	O2—C6—C7—C8	−179.8 (2)
C3—C2—C4—O1	69.5 (3)	C5—C6—C7—C8	1.1 (3)
Br1—C2—C4—O1	−173.2 (2)	C6—C7—C8—C9	0.5 (4)
C1—C2—C4—N1	129.2 (2)	C7—C8—C9—C10	−1.2 (3)
C3—C2—C4—N1	−107.6 (3)	C7—C8—C9—C11	177.4 (2)
Br1—C2—C4—N1	9.8 (3)	C6—C5—C10—C9	1.4 (3)
C4—N1—C5—C10	7.8 (4)	N1—C5—C10—C9	−178.5 (2)
C4—N1—C5—C6	−172.1 (2)	C8—C9—C10—C5	0.2 (3)
C10—C5—C6—O2	178.8 (2)	C11—C9—C10—C5	−178.4 (2)
N1—C5—C6—O2	−1.3 (3)		

### Hydrogen-bond geometry (Å, °)

**Table:**

*D*—H···*A*	*D*—H	H···*A*	*D*···*A*	*D*—H···*A*
O2—H1H···O1^i^	0.85 (1)	1.81 (1)	2.659 (2)	179 (3)
C1—H1C···O2^ii^	0.98	2.53	3.445 (3)	156.
N1—H1N···Br1	0.87 (3)	2.47 (3)	3.031 (2)	123 (2)

Symmetry codes: (i) *x*+1, *y*, *z*; (ii) *x*−1, *y*, *z*.
